# Natural peloids originating from subsea depths of 200 m in the hupo basin, South Korea: physicochemical properties for potential pelotherapy applications

**DOI:** 10.1007/s10653-024-02014-2

**Published:** 2024-06-07

**Authors:** Changyun Park, Jae-Hwan Kim, Woohyun Choi, Daeyoung Kim, Sang-Gun No, Donghoon Chung, Hae-in Lee, Seungbin Seo, Sung Man Seo

**Affiliations:** 1 School of Earth System Science, Kyungpook National University, Daegu, 41566 Republic of Korea; 2 Korea Institute of Geoscience and Mineral Resources, 124 Gwahang-ro, Daejeon, 305-350 Republic of Korea

**Keywords:** Peloid, Natural peloid, Pelotherapy, Hupo basin, Seafloor sediment

## Abstract

The present study firstly reports surface sediment from the subsea depth of 200 m as a potential natural peloid. The fine-silt sediment exhibited a consistent clay mineral composition dominated by illite, chlorite, kaolinite, and diatomite. The most abundant clay mineral was illite/mica, with other minerals loosely packed in a face-to-face orientation. The thermal conductivity, specific heat capacity, and cation-exchange capacity of the sediment were in the range 0.855–0.885 W/m K, 2.718–2.821 J/g °C, and 23.06–32.96 cmol/kg, respectively. The concentrations of most toxic elements in the sediment were considerably lower than the limits set by domestic cosmetic regulations and other international standards. The analyzed samples exhibited similar properties to those of previously reported peloids, thus making them suitable for use in the field of pelotherapy; furthermore, the consistency in data across a wide peloid-distribution area is expected to enable economically viable mining. Future investigations should aim to to evaluate the long-term effects on the skin, the bioavailability of potentially hazardous substances, and the therapeutic efficacy for various skin conditions.

**Supplementary Information:**

The online version contains supplementary material available at 10.1007/s10653-024-02014-2.

## Introduction

Pelotherapy refers to the utilization of natural or maturated peloids for healing and cosmetic purposes; this type of treatment has been extensively studied, particularly in rheumatology and dermatology (Beer et al., 2003; Bellometti et al., 2005; Carretero, 2020; Codish et al., 2005; Evcik et al., 2007; Fioravanti et al., 2007; Forestier et al., 2010; Fraioli et al., 2011; Sukenik et al., 1990). Herein, natural peloids are defined as muds or muddy suspensions that mature in nature at the sites where they occur (Gomes et al., 2013). Notably, natural peloids are typically sampled from easily accessible locations, potentially making them susceptible to quality degradation due to human activities and ecosystem influences (Almeida et al., 2023). To mitigate the short-term and long-term negative impacts on peloids, surface sediments at great depths from the sea floor may be used as alternatives.

The present study focused on assessing the potential utility of the seafloor surface sediment distributed at a depth of 200 m in the Hupo basin of the East Sea, South Korea, as peloid deposits. The Hupo basin is considered a promising area for peloid exploration for several reasons. First, it is geologically isolated from various anthropogenic sources of contamination. Second, the seafloor surface sediments in the Hupo basin have previously been extensively investigated through depositional and geochemical analyses, providing valuable background information for assessing their potential application as peloids (Hong et al., 2022; Khim et al., 2021; Kim et al., 2010; Lee et al., 2015; Son et al., 2022). Third, box/piston core analyses have confirmed that these sediments have a consistent composition across a wide area.

Therefore, the present study aimed to characterize the geological properties of the seafloor surface sediment originating from a previously unexplored depth of 200 m, determine its physicochemical properties, including thermal properties, and assess its suitability for use in pelotherapy through comparison with commercially available peloids from abroad.

## Geological setting

**Fig. 1:**
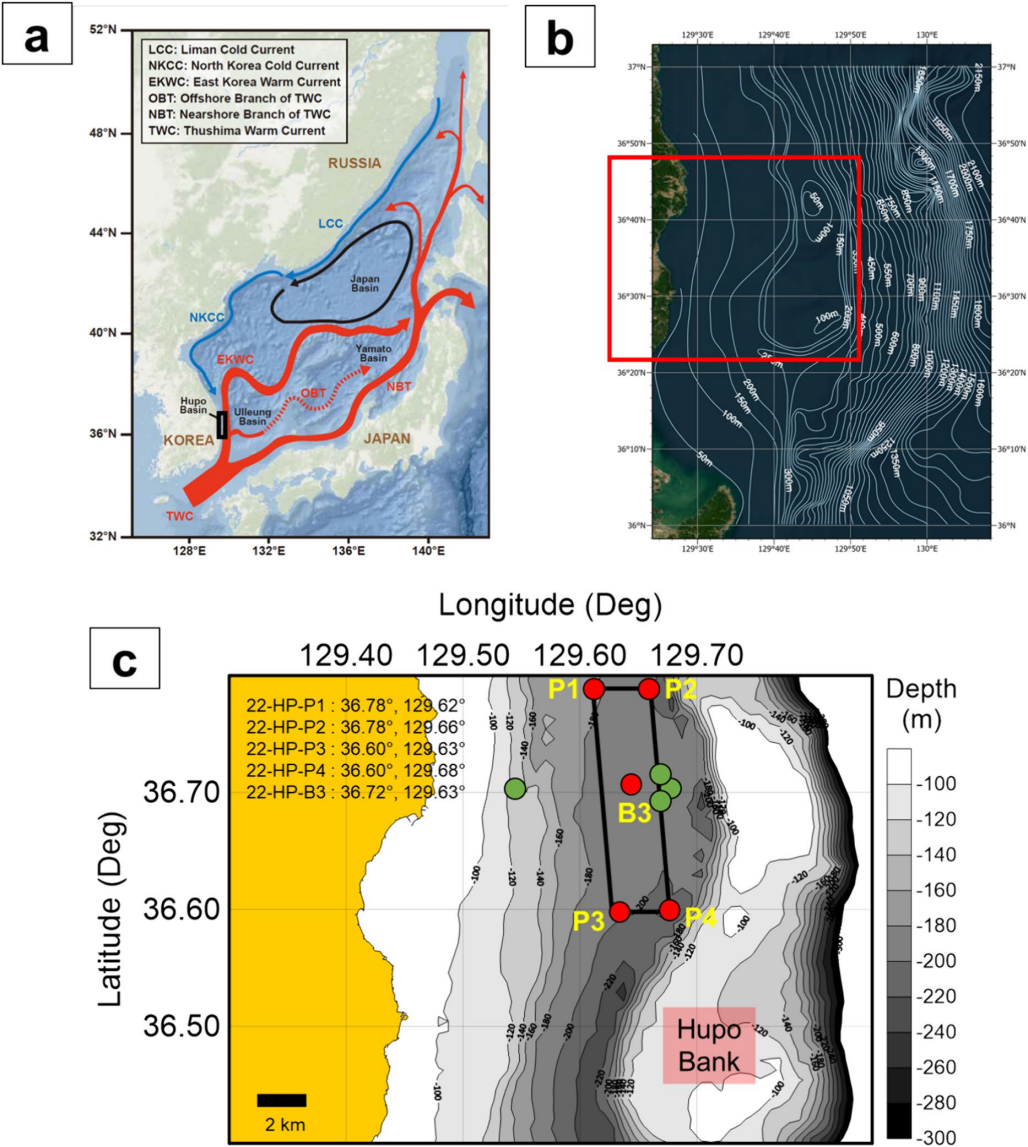
**a** Physiography and surface currents in the East Sea (Japan Sea), **b** bathymetry of the Hupo basin and Hupo Bank, and **c** bathymetry of the study area in the Hupo basin with marked piston/box core locations (red circle). In this study, “P” denotes the piston core, while “B” represents the box core. Sampling was performed by deploying the piston core at the corner points of the rectangular area and box core at the center of the rectangular area to assess the uniformity of surface sediment and distribution area of peloid deposits. The green circles represent prior research locations in the Hupo Basin (Jun et al., 2014; Khim et al., 2021; Lee et al., 2015)

The East Sea (Sea of Japan) is a continental back-arc basin composed of three distinct basins: the Ulleung, Yamato, and Japan basins (Fig. 1a) (Kim et al., 2011). The Ulleung Basin is located in the southwestern region of the East Sea and encompasses several sedimentary basins, including the Pohang–Youngduk, Mukho, and Hupo basins (Kim et al., 2011; Park et al., 2022; Yoon et al., 2014). The Hupo basin occupies the western part of the eastern Korean continental margin, stretching from 35°N to 40°N in latitude (Fig. 1b) (Kim et al., 2015; Yoon et al., 2014). Notably, it is characterized by a half-graben geological structure (Kim et al., 2015, 2018; Yoon & Chough, 1995; Yoon et al., 2014). In accordance with the half-graben structure parallel to the shoreline, the Hupo Bank, a submarine ridge, and the Hupo Trough, a depression, are observed (Fig. 1c). The Hupo Bank extends for approximately 100 km with water depths ranging from 10 to 200 m (Fig. 1c). The Hupo Trough is adjacent to the Hupo Bank and is situated on a wide, flat, and shallow continental margin (less than 200 m deep).

**Fig. 2:**
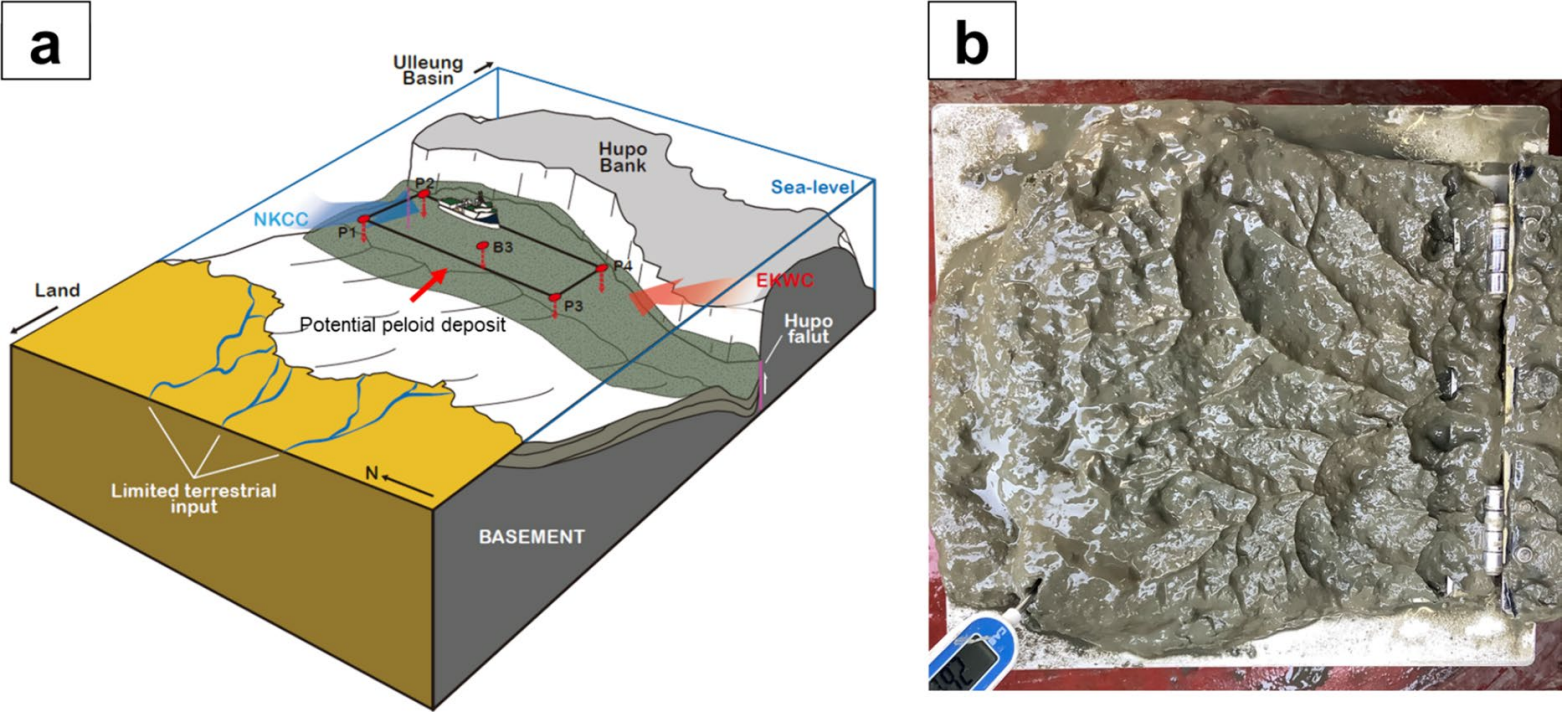
**a** Schematic of the surface sedimentation in the Hupo basin, where sediment deposition is constrained by the geological environment that limits terrestrial sediment input. Hemipelagic sediment is supplied by the warm and high-salinity EKWC flowing from the south to the north and the cold, low-salinity NKCC beneath the EKWC (Hong et al., 2022); **b** Photo of the seafloor surface sediment from the Hupo basin, collected using a box core

The basement rock of the Hupo basin primarily consists of Precambrian gneiss and Jurassic granite, overlaid by Quaternary and Pliocene sediment (Hong et al., 2022). In particular, the sediment covering the uppermost layer (up to ~ 6 m) primarily consists of sandy mud/mud that was intensively deposited during the Late Pleistocene and Holocene; notably, such deposits are most abundantly distributed within Hupo Trough (Fig. 2a and b) (Hong et al., 2022; Jun et al., 2014). The surface sediment in the study area was considered to originate primarily from the transport by ocean currents, particularly the East Korean Warm Current (EKWC) and North Korean Cold Current (NKCC; Fig. 2a) (Hong et al., 2022; Khim et al., 2021) because most rivers capable of transporting sediment to the East Sea are short and small, and terrestrial sediments tend to accumulate within the shallow continental shelf, thus limiting the supply of terrestrial input (Khim et al., 2021).

Kim et al. (2011) have conducted extensive sedimentological and geochemical studies on surface sediments using a 6 m gravity core. Their research demonstrated that, based on a depth of approximately 5 m (^14^C age: 8.2 ka) as a reference point, sedimentation rate, total organic carbon (> 2%), and biogenic opal content (> 8%) increased, whereas grain size decreased toward the sediment surface. These findings suggest that the study area was significantly affected by seawater fluctuations due to abrupt changes in oceanographic conditions, specifically the transition from the Weichselian glacial period in the Pleistocene to the interglacial period in the Holocene. Furthermore, the rise of sea level in the Holocene period has been reported to play a crucial role in stabilizing the conditions of sediment deposition, inducing the deposition of hemipelagic sediment at high rates (~ 56.6–91.0 cm/kyr).

## Sampling and analytical methods

The samples were obtained from the surface sediment at a subsea depth of 200 m in the Hupo basin; the topmost sediment from four piston cores (HP-P1, P2, P3, and P4) and one box core (HP-B3) were used for analysis. The analytical methods are detailed in the supplementary materials (Supplement 1).

## Results and discussions

### Mineralogical and micro-fabric characteristics

**Fig. 3:**
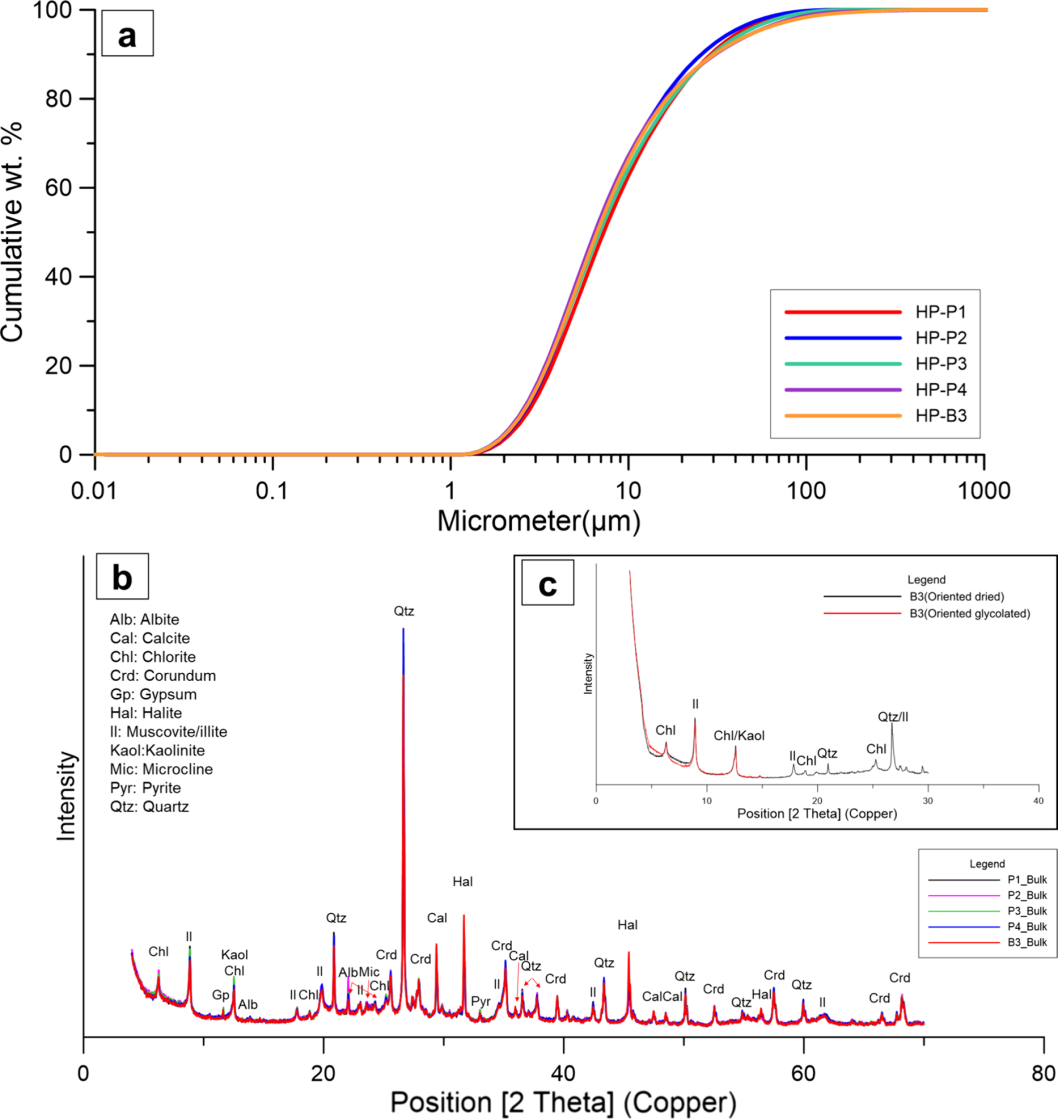
**a** and **b** XRD patterns of the bulk seafloor surface sediment in the Hupo basin and **c** that of oriented dry and glycolated 2022-HP-B3 samples

As shown by the granulometric analysis, surface sediment samples were primarily composed of clay, very fine silt, and fine silt. Their contents were 23.5%–27.5% clay (< 4 μm), 33.1%–34.7% very fine silt (4–8 μm), 18.0%–20.7% fine silt (8–16 μm), 9.9%–13.1% medium silt (16–31 μm), 6.0%–7.3% coarse silt (31–62 μm), and 1.2%–3.4% sand (> 62 μm; Fig. 3a). Compared with other peloids, the relatively small particle size of the samples was due to the geological conditions of the sedimentary environment, where the input of terrestrial sediments was strictly limited (Khim et al., 2021). Furthermore, as previously demonstrated, smaller clay particles are associated with slower cooling (Berbenni, 1965), suggesting that the studied sediment might possess improved thermal properties.

The minerals detected in the samples and their contents are summarized in Table 1, and the relative contents of clay minerals with the < 2 µm particle size are listed in Table 2. The diffraction patterns of the samples are shown in Fig. 3b. This result shows that the mineralogical composition of the five samples were nearly identical, and the consistency observed in the X-ray fluorescence data also supported these findings (Supplement 2). Furthermore, the mineral composition observed is not restricted to this study area but is predominantly characteristic of surface sediments covering the uppermost part of the Hupo Basin. Previous studies on surface sediments in the Hupo Basin demonstrate agreement with our findings regarding mineral composition and particle size distribution at various locations (Fig. 1c) (Jun et al., 2014; Khim et al., 2021; Lee et al., 2015). Additionally, through comparison of surface sediments at depths of 200 m and 120–170 m in the Hupo Basin, it was determined that the uppermost sediments with a minimum thickness of 4 m at both locations originated from hemipelagic sources, exhibiting similar mineralogical and geochemical features (Fig. 1c and 2a) (KIGAM, 2021; Kim et al., 2010). Regarding the reason why similar peloids of the same composition were distributed throughout the Hupo Basin, Hong et al. (2022) reported that the contourite depositional system, originating from the intermittent north–south paleo-NKCC (North Korean Cold Current) in the Hupo graben, caused lateral migration of sediment. These findings suggest that the surface sediments comprising the Hupo Basin possess substantial mineable reserves and offer consistency in materials when utilized as peloids.

**Table 1:** Mineral contents (wt %) in the seafloor surface sediments from the Hupo basin, as determined via XRD

Bulk mineralogy					
	HP-P1	HP-P2	HP-P3	HP-P4	HP-B3
Quartz	17.7	16.2	16	17.3	17
Plagioclase	5.5	6	5.1	5.2	5.1
K-feldspar	2.1	2.5	2.1	2.5	2.4
Muscovite/Illite	13.7	12.5	12	13.1	12.6
Chlorite	4.3	4.8	4.7	4.4	4.3
Kaolinite	1.5	1.8	1.6	1.8	1.4
Amphibole	0.3	0.3	0.3	0.3	0.3
Calcite	3.8	4.1	4.3	4.3	5.7
Halite	3	2.4	4.5	4.2	5.9
Gypsum	trace	n.d	0.5	n.d	0.6
Pyrite	0.7	0.5	0.6	0.5	0.6
Amorphous	47.4	48.9	48.3	46.4	44.1

Note: n.d.: not detected

**Table 2:** Relative contents of clay minerals in the < 2 µm size fraction

Clay speciation	
	HP-B3
Illite	76
Chlorite	17
Kaloinite	7

Phyllosilicate minerals in analyzed samples comprised mica/illite, chlorite, and kaolinite, whereas detrital minerals included quartz, plagioclase, K-feldspar, and amphibole. Authigenic minerals comprised calcite, halite, gypsum, pyrite, and the amorphous phase (e.g., diatomite). X-ray diffraction (XRD) patterns of oriented samples revealed the presence of illite (76%), chlorite (17%), and kaolinite (7%; Table 2). The high crystallinity of the most abundant mineral, illite, indicated the deposition in a warm and humid climate, aligning with the previously mentioned effects of the EKWC during the Holocene climate optimum in the study area (5–7 ka) (Jun et al., 2014; Park et al., 2012; Yi, 2011). According to literature, mineral assemblages containing illite, chlorite, and kaolinite were often used in spa and cosmetic therapies and mud samples with high contents of quartz, carbonates (such as calcite), and illite have high therapeutic applicability (Carretero, 2020; Glavaš et al., 2017). Particularly, illite-dominant sediments exhibited similar mineralogical composition as peloids in the Ria de Aveiro saltpans in Portugal and Sečovlje Salina in Slovenia (Almeida et al., 2023; Glavaš et al., 2017). The presence of a significant amorphous phase is one of the unique mineralogical features of the seafloor surface sediment in the Hupo basin. As presented in Table 1, the amorphous phase was mostly considered to be diatomite composed of opal-A. Notably, the adsorption capacity and microporosity were derived from the morphology of diatomite. First, the high water adsorption capacity of diatomite contributed to the slow cooling rate of the peloid (Kogel, 2006; Veniale et al., 2004). Water retention is important because it is closely related to specific heat. Second, micropores in diatomite could increase the specific surface area (SSA), thereby enhancing the peloid reactivity. The SSA of the samples ranged 33.85–35.88 m^2^/g. This value is within the usable range considering the SSAs of other peloids (Carretero, 2020).

**Fig. 4:**
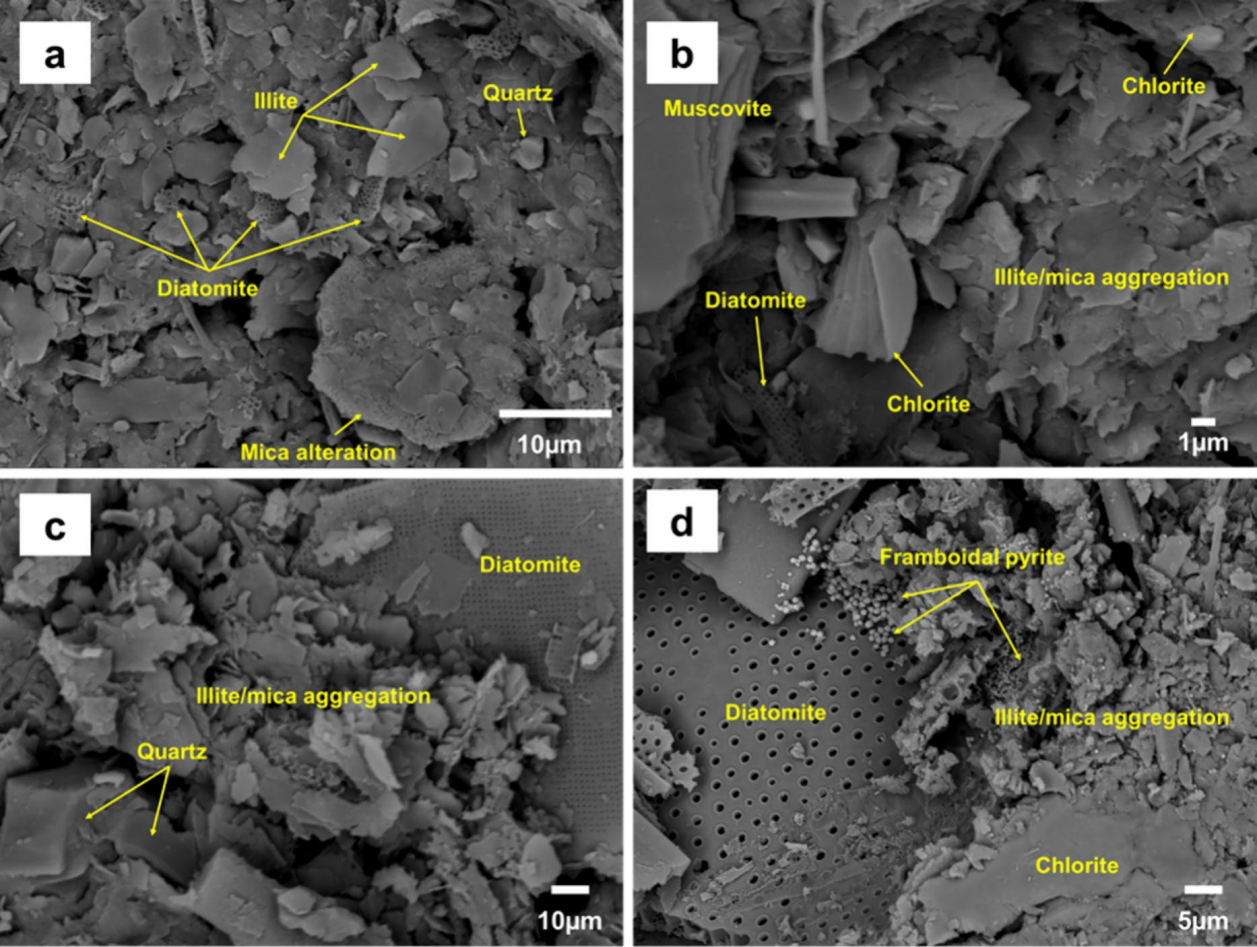
**a**–**d** SEM images of the seafloor surface sediment from different sites in the Hupo basin. These mainly feature platy illite/mica aggregates, diatomite, quartz, and chlorite. Illite/mica, which are most abundantly observed in SEM images are loosely packed with other minerals

To preserve the internal structure of the peloid paste, the paste for analysis was pretreated using a freeze-drying system (Fernández-González et al., 2017). Scanning electron microscopy (SEM) images of the freeze-dried samples demonstrated loosely compacted aggregates and matrix-supported sediment, where few micrometer-sized mineral particles (sub µm; illite, small-sized diatomite, and quartz) were wrapped around tens-of-micrometers-sized mineral grains (feldspar, mica, and large sized diatomite; Fig. 4a–d). The primary clay mineral in the analyzed samples, illite, predominantly exhibited a particle size of < 5 µm and was commonly observed in the form of aggregates of pseudohexagonal platelets. Some individual grains were separated from these aggregates and covered the surfaces of other minerals (Fig. 4a and c). Boundary conditions were characterized by the face–face orientation with seldom-observed face–edge, and illite were stacked in a booklet-like shape (Fig. 4a–c). Chlorite and quartz exhibited particles of varying sizes (ranged from a few micrometers to tens of micrometers) and diverse orientations (Fig. 4b–d).

As mentioned in the XRD results, amorphous silica constituted 44.1–48.9 wt% of the analyzed samples, attributed to diatomite. Diatomite particles exhibited various sizes in the range 10–150 µm, and were frequently observed in all samples (Fig. 4). These particles were predominantly arranged in a connection phase geometrically similar to illite aggregates. The microporosity of diatomite is also worth mentioning. On the side of the microtexture of peloid, high porosity implies improved thermal behavior (Legido et al., 2007), which is expected to compensate for the drawbacks observed in the thermal properties of the loosely compacted fabric and cementation in the samples. Meanwhile, framboidal pyrite was often found in the seafloor surface sediment (Fig. 4d). Although it may release As and thus have harmful effects (Tabelin et al., 2018), the average As content of 7.4 ppm indicates that the As content in framboidal pyrite did not considerably affected the toxic element content.

### Toxic elements

During the therapeutic application of peloids, they come into contact with the skin, potentially exposing individuals to various toxic metals. Despite the skin’s protective function, studies have shown that it can still permit the penetration of chemicals, including heavy metals (Kezic & Nielsen, 2009; Lim et al., 2018), which suggests that dermal contact serves as a route for exposure to heavy metals present in peloids, posing inherent health risks (Bastos et al., 2024). Pb and Cd in the human body has been linked to several severe health conditions, including cancer, kidney disorders, skeletal damage, brain damage, and reproductive failure (Miranzadeh et al., 2023). Cr can cause adverse effects on the respiratory and digestive systems (Shadreck & Mugadza, 2013; Tang et al., 2016). Long-term As accumulation can induce a variety of cancers (skin, lung, liver, and bladder) and Blackfoot disease (Li et al., 2021).

In most countries, including South Korea, heavy metals levels in peloids are not officially regulated. Therefore, to ensure the safety of products containing peloids for therapeutic applications, the peloids in question must be assessed considering the heavy metal content in various regulations related to therapeutic applications. Especially, the ICH-Q3D regulations classify elements that can be toxic at low concentrations into three distinct classes.

Class 1 includes As, Cd, Hg, and Pb, representing substances toxic to humans, which are either restricted or prohibited in pharmaceutical manufacturing.

Class 2A and B encompass elements such as Ni, V, Se, Tl, and Co, which have lower toxicity than Class 1 but should still be restricted in pharmaceuticals because of their potential toxicity.

Class 3 comprises elements such as Cr and Cu, which are toxic substances that should be considered metallic impurities in cosmetic products.

Table 3 presents permissible limits for heavy metals and related standards for various countries, including regulations of cosmetic products in South Korea. The concentrations of toxic elements in the samples were well below the thresholds for cosmetic products in South Korea as well as other standards. Although the Pb content did not reach the limits specified by ICH-Q3D for oral and inhalation routes, simply assessing the content of toxic elements does not suffice to determine the therapeutic appropriateness of peloids. First, evaluating the potential harm of peloids should focus on health risk assessment through dermal bioavailability, rather than relying on the total concentrations of heavy metals based on oral and inhalation pathways. Wang et al. (2022) investigated the dermal bioaccessibility and cytotoxicity of heavy metals in urban soils from Kunming, Yunnan, China, focusing on the health risks of skin exposure to heavy metals such as Cr, As, Cd, Pb, and Cu. The research measured both the total and skin-absorbable concentrations of these metals and evaluated their effects on human keratinocytes (HaCaT cells). Despite finding relatively high total concentrations of heavy metals in soil samples, the bioaccessibility through the skin was generally low. Several studies on the dermal bioavailability of these heavy metals have also been conducted on peloids. Through in vitro permeation experiments using Turkish peloid, the skin permeability of certain heavy metals was observed, and it was also noted that the amount of heavy metals able to migrate through the skin layer after application of the peloids was below acceptable levels (Bastos et al., 2024). Additionally, Carretero et al. (2010) reported that potentially toxic elements were leached only in very low concentrations or not at all by artificial sweat. Heavy metals such as Cu, Ni, Pb, and Zn tended to move from the sweat solution to the peloids, observed at very low concentrations (below 0.1 µg/g). However, despite bioavailability through the skin was generally low as shown above mentioned, the cytotoxicity tests revealed that some soil extracts could significantly affect cell morphology and viability, and induce cell apoptosis (Wang et al., 2022). Several studies have reported comparable findings regarding the toxicological impacts of heavy metals on human cells, particularly emphasizing the induction of cell apoptosis and the alteration of cellular functions upon exposure (Bae et al., 2001; Bishayi & Sengupta, 2006; Carlisle et al., 2000; Zhang et al., 2003). These investigations examine the mechanisms through which metals like arsenic, cadmium, chromium, and lead contribute to oxidative stress, DNA damage, and the activation of apoptosis pathways in various cell types, underscoring the potential health hazards posed by dermal and environmental exposure to these contaminants. The findings from these studies serve as a complicating factor in assessing the potential harm of heavy metals present in peloids. Therefore, the ability of heavy metals to induce oxidative stress, DNA damage, and apoptosis suggests that the assessment of peloid’s safety must consider not just their therapeutic benefits but also the potential risks associated with heavy metal exposure. This necessitates a more nuanced approach to evaluating their health implications, taking into account the bioavailability and toxicological profiles of the contained metals. Secondly, it’s necessary to take into account the form in which heavy metals are present within the peloid. Following the assessment of exchangeable heavy metals fractions using MgCl_2_, the analyzed heavy metals were not found (see Supplement 3). These findings indicate that the quantity of heavy metal that can be adsorbed onto the mineral surface or exist in an oxide form within the pore water is rare. In essence, the amount of heavy metal that actually contacts skin tissue is significantly low compared to the overall concentration, suggesting a potentially positive outcome. KIGAM (2021) reported that, following a 5-day treatment of peloids from the study area with 0.5 M hydrochloric acid, the levels of heavy metals such as Ni and Pb decreased by 44% and 79%, respectively. This implies that these heavy metals are likely present in loosely bound oxide, sulfide, or carbonate minerals. This aligns with observations of framboidal pyrite or calcite occurring as minor minerals within the peloids from the study area (Fig. 4d). Given that heavy metals can be integrated into the clay matrix, from which their extraction is notably challenging (Carretero et al., 2010), this form is presumed to be relatively safe concerning potential health hazards. Nonetheless, due to the health risks associated with the unidentified states of heavy metals, further investigation is necessary. This should include studies on elemental behavior under various redox conditions and pH levels, along with toxicity assessments based on simultaneous exposure or mixing ratios of different compounds.

**Table 3:** Comparison of toxic elements concentrations in Hupo surface sediment using Inductively Coupled Plasma-Mass Spectrometry (ICP-MS) and other standards

Element	Detection limit	22HP-P1	22HP-P2	22HP-P3	22HP-P4	22HP-B3	Average	Domestic cosmetic standards	Barhoumi (2019)	Slovenia No. 68/96^*^	NHPD^†^	USP^‡^ 38	ICH-Q3D^§^
	(µg/g)							(Maximum acceptable level, µg/g)					(Oral PDE^**^, µg/g)
As	0.5	2.3	6.7	8.5	13.1	6.2	7.4	10	8	20	3	5	15(1)
Cd	0.1	0.2	0.3	0.2	0.2	0.2	0.2	5			3		5(1)
Pb	0.5	20.9	20.7	19.7	19.3	24.7	21.1	50	50	85	10	40	5(1)
Se	0.1	1.1	1.2	1.0	0.9	0.6	1.0						150(2B)
Tl	0.05	0.7	0.7	0.7	0.7	0.7	0.7						8(2B)
Ba	1	395.0	392.0	371.0	363.0	383.0	380.8		1300				1400(3)
Cr	1	75.0	75.0	80.0	88.0	71.0	77.8		1100	100			11,000(3)
Cu	0.2	19.9	19.5	20.3	20.4	20.2	20.1		130	60			3000(3)
Ni	0.5	40.3	40.4	41.2	39.4	36.9	39.6		60	50			200(2A)
V	2	98.0	102.0	98.0	97.0	100.0	99.0						100(2A)
Zn	0.5	109.0	112.0	109.0	109.0	109.0	109.6		200	200			
Sr	0.2	184.0	171.0	206.0	214.0	183.0	191.6						
Te	0.1	< 0.1	< 0.1	< 0.1	< 0.1	< 0.1	< 0.1						
Co	0.1	12.2	12.3	13.7	17.3	13.3	13.8			20			50(2A)
Mo	0.2	3.9	3.8	4.4	5.0	3.0	4.0			10			
Hg	1	< 1	< 1	< 1	< 1	< 1	< 1	1			1		30(1)
Sb	0.1	0.8	1.0	0.7	0.7	1.8	1.0	10					

*Slovenia No.68/96: Slovenian legislative concemtrations for soils

^†^NHPD: According to the Natural Health Products Guide of the Canadian Natural and Non-Prescription Health Products Directorate

^‡^USP: United States Pharmacopoeia

^§^ICH-Q3D: Elemental Impurities According to International Council for Harmonization Q3D

**PDE: Permitted Daily Exposure

### Physicochemical properties

Illite and muscovite surfaces are hydrophilic due to an imbalance of negative charges, while diatomite particles serve as effective carriers for microorganisms due to their high porosity and good hydrophilicity (Pan et al., 2020; Yin et al., 2012). Hydrophilic substances dissolved in pore water, along with the distribution characteristics of hydrophilic minerals, mean that the peloid in the study area exhibits hydrophilic properties. In connection with the hydrophilic nature of these peloids, the concentration of exchangeable cations, known as cation-exchange capacity (CEC), is of great significance because exchangeable cations are bioavailable and can reach a patient’s skin through sweat (Tateo & Summa, 2007; Tateo et al., 2009). A high CEC value is desirable because it signifies the prospect of a material to trap potentially harmful elements, which is essential for therapeutic benefits. The ability to exchange cations primarily depends on the clay mineral composition (Aguzzi et al., 2007; Quintela et al., 2015). The CEC values of the five samples investigated here ranged 23.06–32.96 cmol/kg, with all samples except 22-HP-P1 exhibiting a consistent value of approximately 30 cmol/kg (Table 4). According to Meunier et al. (2004), illite, unlike mica, possessed interlayer sites with low or no charge, resulting in a higher CEC value (20–40 cmol/kg) than that of mica (5–10 cmol/kg). The presence of organic matter could increase the CEC value. Considering that the samples used in this study are naturally occurring seafloor sediments, the CEC values were predominantly derived from illite and organic materials. The CEC values of the analyzed samples fell within the range of 11–250 cmol/kg, in comparison to other peloids (Table 4). The CEC values of other peloids already in use as spa products were found to range from 26 cmol/kg to 30 cmol/kg for Peruíbe black mud (Da Silva et al., 2015), from 49 cmol/kg to 61 cmol/kg for a northern Italian spa center (Veniale et al., 2004), from 9 cmol/kg to 32 cmol/kg for a Turkey spa center, and from 30 cmol/kg to 90 cmol/kg in Italian Sardinia deposits (Cara et al., 2000). Because this illite-based peloid has relatively strong resistance to changing pH when applied to the skin (Jozefaciuk, 2002), it is considered to have the advantage of maintaining stable physical properties when using the peloid for therapeutic purposes. However, the studied peloids have a relatively low CEC value compared to others (Table 4), likely because the dominant clay minerals in the study area form an illite-based mineral assembly rather than a smectite-based one, which typically boasts a high CEC (Komar et al., 2015; Pozo et al., 2013). Even though the analyzed samples fall within the CEC range deemed suitable for peloid use, it might be beneficial to enhance the peloid’s physical properties by incorporating minerals like montmorillonite or by maturation process with mineral water.

**Table 4:** Physical and thermal properties of peloid from the Hupo basin and other peloids

	Natural peloid											Peloid (maturation)			
	This study	Italy (Sardinia) Cara et al. (2000)	Spain (Toledo) Cara et al. (2000)	Portuguese (Rebelo et al. (2010)	Spain Casás et al. 2013)	Spain Caridad et al. (2014)	Spain Carretero et al. (2014)	Spain Armijo et al. (2016)	Spain (Granada) García-Villén et al. (2017)	Spain Pozo et al. (2018)	Tunisia Khiari et al. (2019)	France Knorst-Fouran et al. (2012)	Italy Veniale et al. (2004)	Slovenia Glavaš et al. (2017)	Spain Carretero et al. (2010)
*Mineral assemblage*															
Clay-sized mineral	Illite, diatomite, chlorite, kaolinite	Smectite, illite, zeolite	Saponite, smectite, illite	Smectite, illite, kaolinite	Saponite, sepiolite, illite	Hectorite, bentonite	Smectite, illite	Smectite, sepiolite, kaolinite	Smectite, mica	Smectite, sepiolite, illite	Illite, kaolinite, smectite	–	Smectite, illite, kaolinite	Illite, smectite, chlorite, smectite-illite, kaolinite	Smectite, illte, kaolinite, chlorite
*Physical properties*															
pH	7.18 ~ 7.21	–	–	–	–	–	–	–	5.03 ~ 7.82	6.84 ~ 10.26	6.18 ~ 8.01	–	8.3–9.7	7.9–8.5	–
Surface area(m^2^/g)	33.847 ~ 35.877	–	89 ~ 220	7.1 ~ 12.5	109 ~ 110	–	6 ~ 97	–	–	78 ~ 293	–	–	–	–	6–97
Water content (%)	52 ~ 61	60 ~ 79	50 ~ 80	–	65	55 ~ 94	31.43 ~ 76.64	60.3 ~ 65.1	46 ~ 92	41.6 ~ 66.6	–	45.2	44.6–74.7	44–50	31–77
Ash content (%)	34.4 ~ 37.1	–	–	–	–	–	22.73 ~ 64.50	32.7 ~ 35.3	–	–	–	–	–	–	–
Cation exchange capacity (CEC, cmol/kg)	23.06 ~ 32.96	30 ~ 88	82.7 ~ 84	68 ~ 73	–	–	11 ~ 112	–	–	71 ~ 80	4.91 ~ 18.75	–	49–61	162–250	11–112
Density (kg/m^3^)	1645	–	–	–	1320 ~ 1330	1040 ~ 1420	1114 ~ 1562	–	–	–	–	1204	–	1478–1516	–
*Thermal properties*		–	–	–	–	–	–	–	–	–	–	–	–	–	–
Thermal conductivity(W/m∙K)^†^	0.854 ~ 0.885	–	–	–	0.772 ~ 0.784	0.601 ~ 0.804	0.40 ~ 0.53	–	–	–	–	0.72	–	0.835–0.859	0.40–0.53
Specific heat capacity (J kg^−1^ K^−1^)	2718 ~ 2821	2560 ~ 3410	2760 ~ 3540	1700 ~ 2600	2890 ~ 2911	–	2970 ~ 3800	2762 ~ 3106	3040 ~ 3860	2427 ~ 3308	–	3240	2040 ~ 3230	2300 ~ 2550	2970 ~ 3800
Thermal diffusivity(10^–7^ m^2^s^−1^)	1.84 ~ 1.98	–	–	–	–	–	1.15 ~ 1.79	–	–	–	–	1.876	–	2.21–2.46	–
Granulometry*(%)		–	–	–	–	–	–	–	–	–	–	–	–	–	–
Clay (< 4 µm)	23.51 ~ 27.52	–	18.9 ~ 45.2	17 ~ 20	77.1 ~ 85.2	–	11.3 ~ 59.0	17.8 ~ 31.43	–	10 ~ 15	–	–	73–81	4–6	11–59
Very fine silt (4–8 µm)	33.07 ~ 35.20	–	–	3 ~ 25	14.8 ~ 21.5	–	40.2 ~ 77.9	65.72 ~ 70.73	–	80	–	–	–	66–80	40–78
Fine silt (8–16 µm)	18.04 ~ 20.68	–	–			–			–		–	–	–	–	–
Medium silt (16–31 µm)	9.87 ~ 13.07	–	–			–			–		–	–	–	–	–
Coarse silt (31–62 µm)	6.04 ~ 7.29	–	–			–			–		–	–	–	–	–
Sand (> 62 µm)	1.23 ~ 3.36	1.1 ~ 32.4	0.4 ~ 6.7	55 ~ 80	0 ~ 1.5	–	0.4 ~ 18.7	0.14 ~ 11.29	–	5 ~ 10	–	–	–	14–30	0.4–19

${D}_{f}=\frac{\lambda }{\rho \bullet cp}$ Thermal diffusivity (D_f_), Thermal conductivity (λ), density (ρ), and specific heat capacity (cp)

*Wentworth (1922) grain size classification

^†^The thermal diffusivity (D) was determined using the following equation (Casás et al., 2013)

Specific heat and thermal conductivity are the crucial factors determining the ability of a solid to retain heat over a specified period, with higher specific heat and lower thermal conductivity corresponding to better heat retention. As reported in Table 4, the thermal conductivities of the samples were 0.855–0.885 W/m K. This value is somewhat higher than the thermal conductivity values reported for smectite-based materials (Table 4). These values are highly consistent with the thermal conductivity values of non-smectite-based peloids, as observed by Glavaš et al. (2017), indicating that the most significant factor determining thermal conductivity is the speciation of clay minerals. The specific heat capacity of the solid part of samples, as determined using differential scanning calorimetry, were within the range of approximately 0.780–1.025 J/g °C. This range is consistent with the specific heat capacities of peloids composed of bentonite, sepiolite, smectite, or kaolinite (Barhoumi et al., 2019). To be considered for use in thermotherapy (for analgesic and anti-inflammatory effects), specific heat capacities of peloid pastes must be first determined. Two equations were used to calculate the specific heat capacity of the peloid paste. Armijo (1991) suggested calculating the specific heat capacity of a peloid paste based on the solid content (*S*) and water content (*W*) in Eq. (1).

1 $$Cp = {1}.{26}0{23} + 0.0{2926}(W){-}0.00{628}(S) + 0.0000{63}(W)(S)$$

Casás et al. (2011) proposed Eq. (2) for calculating the percentages of the solid fraction (*S*) and water fraction (*W*) in the peloid.

2 $$Cp = {1}00S\% \cdot Cp_{{{\text{solid}}}} + W\% \cdot Cp_{{{\text{water}}}}$$

Herein, the solid and water fractions used in Eqs. (1) and (2) were determined using an ash analyzer, and the specific heat capacity of water was considered to be 4.179 J/g °C. The specific heats calculated using Eqs. (1) and (2) were very similar, ranging from 2.718 J/g °C to 2.821 J/g °C and from 2.884 J/g °C to 2.962 J/g °C, respectively. Furthermore, the calculated thermal diffusivity values are in the range 1.84∙10^−7^–1.98∙10^−7^ m2/s (Table 4). The specific heat values of the samples were similar to those previously reported for peloid pastes, suggesting that the investigated sediment possesses suitable thermal properties for use as a spa peloid (Table 4).

## Conclusions and future studies

Seafloor surface sediment collected at a depth of 200 m from multiple sites in the Hupo basin primarily comprised fine silt–sized pelagic minerals and various authigenic minerals. The most distinctive mineralogical feature representing these sediments was the dominance of illite and high diatomite content. Investigation of the thermal properties (thermal conductivity, specific heat capacity, and thermal diffusivity), physicochemical properties (SSA, solid/water ratio, and CEC), and toxic element contents in the samples indicate the high potential of these sediments as pelotherapy materials. Furthermore, the consistency in data across a wide area suggests that the study area holds promise for mining peloid deposits. However, since this study concentrated on the physicochemical properties of peloids from the Hupo Basin, it did not examine their positive or negative effects on the skin. Therefore, future research should involve conducting tests to evaluate the bioavailability of potentially harmful substances and the therapeutic impacts on various skin conditions, to ensure the safe and effective use of peloids.

### Supplementary Information

Below is the link to the electronic supplementary material.

Supplementary file1 (DOCX 29 KB)

Supplementary file2 (XLSX 11 KB)

Supplementary file3 (XLSX 12 KB)

## Data Availability

Data will be made available on request.
