# Supplementary material for: Natural peloids originating from subsea depths of 200 m in the hupo basin, South Korea: physicochemical properties for potential pelotherapy applications
Source: Environ Geochem Health. 2024 Jun 7;46(7):240. doi: 10.1007/s10653-024-02014-2 (PMC11161544; doi:10.1007/s10653-024-02014-2)
Supplement: Supplementary file 1 — Supplementary file1 (DOCX 29 KB) [file 10653_2024_2014_MOESM1_ESM.docx]

**Supplementary material**

1. **Analytical method**

**1.1. Random-oriented X-ray diffraction (XRD) and oriented XRD**

Five samples were analyzed via quantitative XRD analysis, and one sample was investigated for clay speciation. For the quantitative XRD analysis, a portion of each sample was mixed with corundum and packed into a standard holder. Corundum was added as an internal standard. For clay speciation, a portion of the sample was dispersed in distilled water and the <4 µm size fraction was separated through gravity settling of particles in suspension. An oriented slide of the <4 µm size fraction was prepared by placing a portion of the suspension onto a glass slide. The oriented slide was analyzed after drying and treatment with ethylene glycol.

The XRD analysis was performed on a Bruker D8 Endeavor diffractometer equipped with a Cu X-ray source operated under the following conditions: 40 kV and 40 mA, 2θ = 5°–70° for random specimens and 3°–30° for oriented specimens, step size of 0.02°, time per step of 0.5 s, fixed divergence slit with an angle of 0.30°, and sample rotation of 15 rpm. The PDF4/Minerals ICDD database was used for mineral identification. The quantities of the crystalline mineral phases were determined using the Rietveld method, which is based on the calculation of the full diffraction pattern from crystal structure data. The amounts of crystalline minerals were recalculated based on a known percent of corundum, and the remainder to 100% was considered amorphous material. The relative proportions of clay minerals in the <4 µm fraction were calculated using the ratios of their basal-peak areas.

**1.2. Instrumental neutron activation analysis (INAA) and inductively coupled plasma mass spectrometry (ICP-MS) for contents of trace elements**

**INAA**

The methodology referred to as INAA relies on the detection of gamma radiation generated in a sample through neutron irradiation. In this process, 30 g of the sample was sealed in a polyethylene vial and subjected to irradiation alongside flux wires at a thermal neutron flux of 7 × 1,012 ncm^−2^s^−1^. After a 7 day waiting period to allow for the decay of Na-24, the samples were examined using a high-purity Ge detector with a resolution higher than 1.7 keV for the 1,332 keV Co-60 photopeak. The decay-corrected activities were then compared with the calibration values obtained using multiple certified international reference materials, aided by the flux wires and control standards. One standard analysis was conducted for every 11 samples, and one blank sample was analyzed per work order. Duplicate analyses were performed whenever additional sample material was available.

**Near total Digestion–ICP & near total Digestion–ICP-MS**

For the "near total" Digestion process, a 0.25 g sample was digested using four acids, starting with hydrofluoric acid, followed by a combination of nitric and perchloric acids. The digestion was performed using a precisely programmed heating regime, which involved several ramping and holding cycles, eventually yielding the samples with incipient dryness. After reaching incipient dryness, the samples were redissolved in aqua regia. Subsequently, the samples were analyzed using an ICP system. The multiacid digestion solution mentioned above was diluted and then analyzed using ICP-MS to detect additional elements.

**1.3. CEC (cation-exchange capacity)**

For CEC analysis, marine peloid samples were prepared by mixing 20 g of each sample to ensure representativeness and freeze-drying at −60°C. The freeze-dried samples were then finely ground to prevent aggregation. After the sample preparation, a 0.1 M ammonium acetate solution was prepared, mixed with the samples at a ratio of 1:10 using a stirrer, and filtered to measure CEC. CEC was calculated by summing the base cations that are quantified after extracting samples with ammonium acetate solution (pH = 7)

**1.4 Extraction of exchangeable heavy metal**

To examine how much of the heavy metal in a sample is easily replaceable, a specific method was used. First, a chemical solution made of 0.5M magnesium chloride (MgCl_2_) was prepared. Then, 1 gram of the sample, which is a type of mud used for therapeutic purposes (peloid) and has been finely crushed to a very small size (80 mesh or smaller), was mixed with 8 milliliters of this MgCl_2_ solution. The pH of the solution was adjusted to be neutral (pH 7), and this mixture was shaken for 10 minutes to ensure a thorough reaction. After shaking, the mixture was placed in a centrifuge, which is a machine that spins at high speeds, at 2000 revolutions per minute (rpm) for 15 minutes. This spinning caused the solid part of the sample to separate from the liquid. The liquid part above the solid, known as the supernatant, was then carefully collected. This collected liquid was analyzed using a technique called Inductively Coupled Plasma Optical Emission Spectroscopy (ICP-OES) to determine the amount of heavy metal present.

**1.5. Thermal properties**

**1.5.1. Thermal conductivity**

The thermal conductivity measurements were conducted using the Decagon KD2 Pro thermal property analyzer device (Decagon Devices Inc., Pullman, WA, USA). This method is based on a transient hot-wire technique, involving a single-needle sensor inserted into the sample. Each measurement took 15 min in total, where 5 min was dedicated to achieving temperature stability, followed by 10 min of heating using controlled current intensity.

**1.5.2. Specific heat capacity**

The specific heat of each sample in its powder form was determined using differential scanning calorimetry (DSC 8,000, Perkin Elmer). Approximately 3–4 mg of samples was used for each measurement, the heating rate was set at 3°C/min, and the temperature range was set to 36°C–45°C.

**1.6. Microstructure**

To preserve the internal texture of the sample, we freeze-dried (Fernández-González et al., 2017) them. The experimental procedure was as follows. Initially, the samples were rapidly frozen at temperatures ranging from −100°C to −120°C. Subsequently, the frozen samples were subjected to immediate lyophilization using the freeze–dry system. The lyophilized samples were then fractured to obtain freshly cut surfaces of the peloid’s interior, followed by their fixation to aluminum stubs. In the next step, the samples were coated with a layer of gold, ranging from 10 to 15 nm in thickness. Finally, the samples were examined through scanning electron microscopy (JEOL JXA-IHP200F) at an acceleration voltage of 15–20 kV with energy-dispersive X-ray spectroscopy.

**2. References**

Fernández-González, M.V. et al., 2017. Physical properties of peloids prepared with medicinal mineral waters from Lanjarón Spa (Granada, Spain). Applied Clay Science, 135: 465-474.
